# Synthesis of Some New Mono- and Bis-Polycyclic Aromatic Spiro and Bis-Nonspiro-β-Lactams

**DOI:** 10.3390/molecules15010515

**Published:** 2010-01-22

**Authors:** Aliasghar Jarrahpour, Edris Ebrahimi

**Affiliations:** Department of Chemistry, College of Sciences, Shiraz University, Shiraz, 71454, Iran; E-Mail: edris.ebrahimi@gmail.com (E.E.)

**Keywords:** mono- and bis-spiro-β-lactam, polycyclic aromatic imine, 9H-xanthene-9-carboxylic acid, phenoxyacetic acid, [2+2] cycloaddition, tosyl chloride

## Abstract

Some new mono-and bis-polycyclic aromatic spiro-β-lactams and bis-non spiro-polycyclic aromatic β-lactams have been synthesized from imines derived from anthracene-9-carbaldehyde, 2-naphtaldehyde and a ketene derived from 9H-xanthene-9-carboxylic acid and phenoxyacetic acid by a [2+2] cycloaddition reaction. The cycloadducts were characterized by spectral data, including ^1^H-NMR, ^13^C-NMR, IR and elemental analyses. The configurations of some of these mono-spiro-β-lactams were established by X-ray crystal analysis.

## 1. Introduction 

β-Lactams, being a structural unit found in the most widely used antibiotics [1], have occupied a basic position in medicinal chemistry for almost a century now. With the microbes responding to the traditional antibiotics through β-lactamases, the need for novel antibiotics prevails, making synthesis of newer β-lactams ever more important. In addition to their use as antibiotics, β-lactams are increasingly being used as synthons for other biologically important molecules [2,3,4,5,6,7,8,9,10,11]. β-Lactams have been found to act as cholesterol acyl transferase inhibitors [12], thrombin inhibitors [13], human cytomegalovirus protease inhibitors [14], matrix metalloprotease inhibitors [15], cysteine protease [16], and apoptosis inductors [17]. Spirocyclic β-lactams have attracted attention as they have been shown to be β-turn mimetics [18,19] and precursors for α,α-disubstituted β-amino acids [20]. The chartelline has a spiro-β-lactam moiety in its structure marine natural products [21]. It has been found that spiro-β-lactams act as poliovirus and human rhinovirus 3C-proteinases inhibitors [22]. These compounds are mostly synthesized by cycloaddition to an exocyclic bond. Several syntheses of spiro-β-lactams have been reported [23,24,25,26,27,28,29,30,31,32,33,34,35,36,37,38,39,40,41,42]. Polycyclic aromatic β-lactams have shown anticancer and other biological activities [43,44,45]. Some polyaromatic β-lactams have been reported to have polyaromatic ring in part of imines that prepared by Staudinger reaction [46]. Therefore in continuation of our work on the synthesis of novel β-lactams [47,48,49], we present here for the first time the results obtained in the synthesis of mono- and bis-polyaromatic spiro and nonspiro-β-lactams using a modified Staudinger reaction.

## 2. Results and Discussion 

**Scheme 1:**
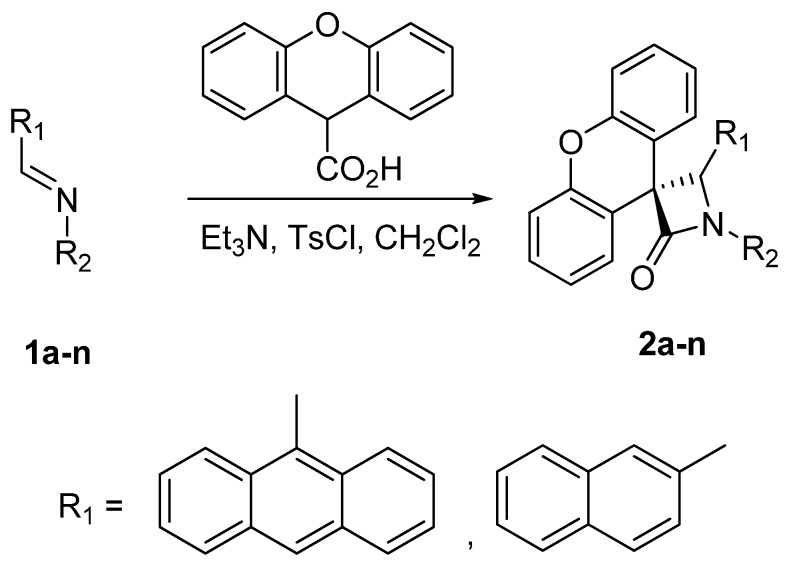
Synthesis of polycyclic aromatic spiro-β-lactams **2a–n**.

Treatment of anthracene-9-carbaldehyde or 2-naphtaldehyde with different primary amines in refluxing ethanol afforded the polycyclic aromatic imines **1a–n** [50,51,52,53]. These Schiff bases were then treated with 9*H*-xanthene-9-carboxylic acid in the presence of triethylamine and tosyl chloride to afford polycyclic aromatic spiro-β-lactams **2a–n** as single diastereomers (Scheme 1). The reaction progress was monitored by TLC and the presence of a new compound was confirmed. In addition, the cycloadducts were characterized by spectral analysis. For **2a** the IR spectrum showed the characteristic absorption of a β-lactam carbonyl at 1,747 cm^−1^. The ^1^H-NMR spectrum exhibited the methoxy protons as a singlet at 3.64 ppm, the β-lactam H-4 proton as a singlet at 6.29 and aromatic protons as a multiplet at 6.39–8.82. The ^13^C-NMR spectrum exhibited the C-3 (spiro carbon) at 64.3. The results for other polycyclic aromatic spiro-β-lactams **2a–n** are shown in Table 1.

**Scheme 2:**
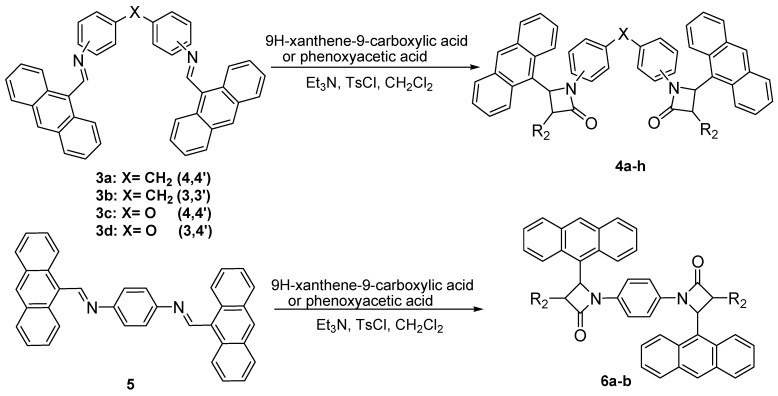
Synthesis of bis-spiro- and bis-nonspiro- polycyclic aromatic β-lactams **4a–h** and **6a–b**.

Then we decided to synthesize bis-spiro- and bis-nonspiro- polycyclic aromatic β-lactams **4a–d**, **6b** and **4e–h**, **6a** from bis-imines **3a–d** and **5** [53] (Scheme 2). The structures of **4a–h** and **5a–b** are shown in Table 2. The cis-trans stereochemistry of 2-azetidinones **4e–h** and **6a** were deduced from the coupling constant of H-3 and H-4, which was calculated to be *J*_3,4_ = 4–9 Hz for the *cis* and *J*_3,4_ = 1–3 Hz for the *trans* stereoisomers.

**Figure 1:**
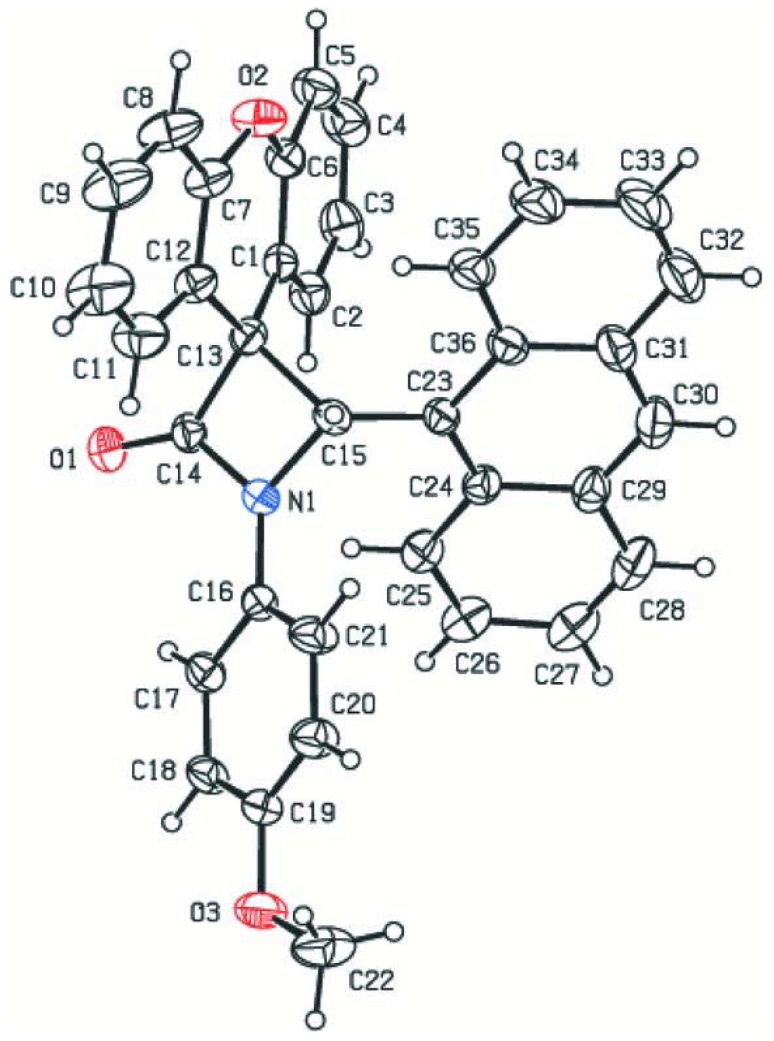
X-ray crystal structure of **2a**.

The X-ray crystallography of **2a** (Figure 1) confirmed the proposed spiro configuration at C3 [54,55,56,57,58,59]. The X-ray analysis also showed that the β-lactam ring is planar and it is perpendicular to xanthene ring. The anthracene ring has dihedral angles of 51.8° with the β-lactam ring. 

## 3. Experimental Section 

### 3.1. General

All needed chemicals were purchased from the Merck, Fluka or Acros chemical companies. All reagents and solvents were dried prior to use according to standard methods [60]. IR spectra were run on a Shimadzu FT-IR 8300 spectrophotometer. ^1^H-NMR and ^13^C-NMR spectra were recorded in DMSO-d_6_ or CDCl_3_ using a Bruker Avance DPX instrument (^1^H-NMR 250 MHz, ^13^C-NMR 62.9 MHz). Chemical shifts were reported in parts per million (δ) downfield from TMS. All of the coupling constants (*J*) are in hertz. The mass spectra were recorded on a Shimadzu GC-MS QP 1000 EX instrument. Elemental analyses were run on a Thermo Finnigan Flash EA-1112 series. Melting points were determined in open capillaries with Buchi 510 melting point apparatus. Thin-layer chromatography was carried out on silica gel F254 analytical sheets obtained from Fluka. Column chromatography was performed on Merck Kiesel gel (230–270 mesh).

### 3.2. General Procedure for Preparation of Schiff Bases ***1a–n***

A mixture of an aromatic amine (1.00 mmol) and anthracene-9-carbaldehyde or 2-naphtaldehyde (1.00 mmol) was refluxed in ethanol (20 mL) for 1–5 hours. After cooling, the pure Schiff bases were separated as crystals. Some of these were recrystallized from ethanol.

*(E)-N-(Anthracen-10-ylmethlene)-4-methxybenzenamine* (**1a**): Yellow powder crystal (yield 91%); Mp: 140–148 °C; IR (CHCl_3_, cm^−1^): 1,608 (C=N); ^1^H-NMR δ (ppm): 3.88 (OMe, s, 3H), 6.52–7.64 (ArH, m, 13H), 9.80 (CHN, s, 1H). Analysis calculated for C_22_H_17_NO: C, 84.86; H, 5.50; N, 4.50%. Found: C, 84.83; H, 5.55; N, 4.48%.

*(E)-N-(Anthracen-10-ylmethylene)-3-methoxybenzenamine* (**1b**): Yellow powder crystal (yield 84%); Mp: 100–102 °C; IR (CHCl_3_, cm^−1^): 1,608 (C=N); ^1^H-NMR δ (ppm): 3.87 (OMe, s, 3H), 6.89–8.74 (ArH, m, 13H), 9.66 (CHN, s, 1H). Analysis calculated for C_22_H_17_NO: C, 84.86; H, 5.50; N, 4.50%. Found: C, 84.83; H, 5.55; N, 4.48%.

*(E)-N-(Anthracen-10-ylmethylene)-2-methoxybenzenamine* (**1c**): Orange powder crystal (yield 76%); Mp: 94–96 °C; IR (CHCl_3_, cm^−1^): 1,620 (C=N); ^1^H-NMR δ (ppm): 6.73–8.75 (ArH, m, 13H), 9.66 (CHN, s, 1H). Analysis calculated for C_22_H_17_NO: C, 84.86; H, 5.50; N, 4.50%. Found: C, 84.83; H, 5.55; N, 4.48%.

*(E)-N-(Anthracen-10-ylmethylene)benzenamine* (**1d**): Yellow powder crystal (yield 76%); Mp: 102–104 °C; IR (KBr, cm^−1^): 1,608 (C=N); ^1^H-NMR δ (ppm): 6.61–8.96 (ArH, m, 14H), 9.63 (CHN, s, 1H). Analysis calculated for C_21_H_15_N: C, 89.65; H, 5.37; N, 4.98%. Found: C, 89.61; H, 5.41; N, 4.95%.

*(E)-N-(Anthracen-10-ylmethylene)-4-chlorobenzenamine* (**1e**): Yllow crystal (yield 51%); Mp: 182–184 °C; IR (KBr, cm^−1^): 1,623 (C=N); ^1^H-NMR δ (ppm): 6.64–8.72 (ArH, m, 13H), 9.61 (CHN, s, 1H). Analysis calculated for C_21_H_14_ClN: C, 79.87; H, 4.47; N, 4.44%. Found: C, 79.85; H, 4.50; N, 4.49%.

*(E)-N-(Anthracen-10-ylmethylene)-3-nitrobenzenamine* (**1f**): Orange crystal (yield 67%); Mp: 166–168 °C; IR (KBr, cm^−1^): 1,666 (C=N); ^1^H-NMR δ (ppm): 6.62–8.96 (ArH, m, 13H), 9.66 (CHN, s, 1H). Analysis calculated for C_21_H_14_N_2_O_2_: C, 77.29; H, 4.32; N, 8.58%. Found: C, 77.35; H, 4.38; N, 8.60%.

*(E)-N-(Anthracen-10-ylmethylene)-2-ethylbenzenamine* (**1g**): Yellow crystal (yield 60%); Mp: 182–184 °C; IR (CHCl_3_, cm^−1^): 1,620 (C=N); ^1^H-NMR δ (ppm): 1.27 (CH_2_, t, 2H, *J* = 7.5), 2.89 (CH_3_, q, 4H, *J* = 7.5), 6.82–8.80 (ArH, m, 13H), 9.55 (CHN, s, 1H). Analysis calculated for C_23_H_19_N: C, 89.28; H, 6.19; N, 4.53%. Found: C, 89.32; H, 6.25; N, 4.55%.

*(E)-N-(Anthracen-10-ylmethylene)-3-bromobenzenamine* (**1h**): Yellow crystal (yield 77%); Mp: 122–124 °C; IR (KBr, cm^−1^): 1,620 (C=N); ^1^H-NMR δ (ppm): 6.84–8.71 (ArH, m, 13H), 9.60 (CHN, s, 1H). Analysis calculated for C_21_H_14_BrN: C, 70.01; H, 3.92; N, 3.89%. Found: C, 70.11; H, 3.98; N, 3.84%.

*(E)-N-(Anthracen-10-ylmethylene)-2,4-dimethoxybenzenamine* (**1i**): Orange crystal (yield 88%); Mp: 160–162 °C; IR (CHCl_3_, cm^−1^): 1,616 (C=N); ^1^H-NMR δ (ppm): 3.75, 3.98 (2OMe, s, 6H), 6.51–8.73 (ArH, m, 12H), 9.92 (CHN, s, 1H). Analysis calculated for C_23_H_19_NO_2_: C, 80.92; H, 5.61; N, 4.10%. Found: C, 80.96; H, 5.66; N, 4.17%.

*(E)-N-(Anthracen-10-ylmethylene)-3,4-dimethoxybenzenamine* (**1j**): Orange crystal (yield 90%); Mp: 160–162 °C; IR (CHCl_3_, cm^−1^): 1,605 (C=N); ^1^H-NMR δ (ppm): 3.88, 3.91 (2OMe, s, 6H), 6.88–8.68 (ArH, m, 12H), 9.92 (CHN, s, 1H). Analysis calculated for C_23_H_19_NO_2_: C, 80.92; H, 5.61; N, 4.10%. Found: C, 80.96; H, 5.66; N, 4.17%.

*(E)-N-(Anthracen-10-ylmethylene)cyclohexanamine* (**1k**): Orange crystal (yield 92%); Mp: 132–134 °C; IR (CHCl_3_, cm^−1^): 1,639 (C=N); ^1^H-NMR δ (ppm): 1.34–3.56 (cyclohexyl, m, 11H), 7.41–8.43 (ArH, m, 9H), 9.34 (CHN, s, 1H). Analysis calculated for C_21_H_21_N: C, 87.76; H, 7.36; N, 4.87%. Found: C, 87.71; H, 7.33; N, 4.91%.

*(E)-N-(Anthracen-10-ylmethlene)naphthalene-1-amine* (**1l**): Yellow crystal (yield 60%); Mp: 142–144° C; IR (CHCl_3_, cm^−1^): 1,624 (C=N); ^1^H–NMR δ (ppm): 6.67–8.94 (Ar-H, m, 16H), 8.80 (CHN, s, 1H). Analysis calculated for C_25_H_17_N: C, 90.60; H, 5.17; N, 4.23%. Found: C, 90.66; H, 5.20; N, 4.28%.

*(E)-N-(Naphthalen-2-ylmethylene)naphthalen-1-amine* (**1m**): Green crystal (yield 78%); Mp: 129–130° C. IR (CHCl_3_, cm^−1^): 1,624 (C=N); ^1^H–NMR δ (ppm): 6.73–8.63 (Ar-H, m, 14H), 8.94 (CHN, s, 1H). Analysis calculated for C_25_H_17_N: C, 90.60; H, 5.17; N, 4.23%. Found: C, 90.66; H, 5.20; N, 4.28%.

*(E)-4-Methoxy-N-(naphthalen-2-ylmethylene)benzenamine* (**1n**): Silver powder crystal (yield 80%); Mp: 118–120° C; IR (CHCl_3_, cm^−1^): 1,620 (C=N); ^1^H-NMR δ (ppm): 3.78 (OMe, s, 3H), 6.90–8.13 (ArH, m, 11H), 8.60 (CHN, s, 1H). Analysis calculated for C_18_H_15_NO: C, 82.73; H, 5.79; N, 5.36%. Found: C, 82.75; H, 5.83; N, 5.32%. 

### 3.3. General Procedure for Preparation of Bis-Schiff Bases ***3a–d*** and ***5***

A mixture of anthracene-9-carbaldehyde (1.00 mmol) and bisamine (0.50 mmol) was refluxed in ethanol (20 mL) for (1–5) h. After cooling, the pure Schiff bases were separated as crystals. Some of these were recrystallized from ethanol.

*(E)-4-(E)-4-[(E)-Anthracen-10-ylmethyleneamino)benzyl]-N-(anthracen-10-ylmethylene)benzene-amine* (**3a**): Yellow powder (yield 96%); Mp: 216–218 °C; IR (KBr, cm^−1^): 1,623 (C=N); ^1^H-NMR δ (ppm): 4.15 (CH_2_, s, 2H), 6.68–9.70 (ArH, m, 26H), 10.02 (CH=N, s, 2H); Analysis calculated for C_43_H_30_N_2_: C, 89.86; H, 5.26; N, 4.87%. Found: C, 89.81; H, 5.22; N, 4.83%.

*(E)-3-(E)-3-[(E)-Anthracen-10-ylmethyleneamino)benzyl]-N-(anthracen-10-yl-methylene)benzenenamine* (**3b**): Yellow powder (yield 95%); Mp: 184–186 °C; IR (KBr, cm^−1^): 1,623 (C=N); ^1^H-NMR δ (ppm): 4.19 (CH_2_, s, 2H), 7.24–8.92 (ArH, m, 26H), 9.73 (CH=N, s, 2H); Analysis calculated for C_43_H_30_N_2_: C, 89.86; H, 5.26; N, 4.87%. Found: C, 89.81; H, 5.22; N, 4.83%.

*(E)-N-(Anthracen-10-yl-methylene)-4-(4-[(E)-anthracen-10-ylmethyleneamino]phenoxy)benzene-amine* (**3c**): Orange powder (yield 96%); Mp: 202–204 °C; IR (KBr, cm^−1^): 1,608 (C=N); ^1^H-NMR δ (ppm): 6.73–8.78 (ArH, m, 26H), 9.60 (CH=N, s, 2H); Analysis calculated for C_42_H_28_N_2_O: C, 87.47; H, 4.89; N, 4.86%. Found: C, 87.51; H, 4.95; N, 4.81%.

*(E)-N-(Anthracen-10-ylmethylene)-3-(4-[(E)-anthracen-10-ylmethyleneamino]phenoxy)benzeneamine* (**3d**): Yellow powder (yield 88%); Mp: 216–218 °C; IR (KBr, cm^−1^): 1,620 (C=N); ^1^H-NMR δ (ppm): 6.20–9.01 (ArH, m, 26H), 9.71 (CH=N, s, 2H); Analysis calculated for C_42_H_28_N_2_O: C, 87.47; H, 4.89; N, 4.86%. Found: C, 87.51; H, 4.95; N, 4.81%.

*(N^1^E,N^4^E)-N^1^,N^4^-bis(Anthracen-10-ylmethylene)benzene-1,4-diamine* (**5**): Yellow powder (yield 95%); Mp: > 240 °C; IR (KBr, cm^−1^): 1,604 (C=N); ^1^H-NMR δ (ppm): 7.25–8.84 (ArH, m, 22H), 9.81 (CHN, s, 2H). Analysis calculated for C_36_H_24_N_2_: C, 89.23; H, 4.99; N, 5.78%. Found: C, 89.28; H, 5.05; N, 5.73%.

### 3.4. General Procedure for the Synthesis of Polycyclic Aromatic Spiro-β-Lactams ***2a–n***

A mixture of Schiff base (1.00 mmol), triethylamine (5.00 mmol), 9*H*-xanthene-9-carboxylic acid (1.50 mmol) and tosyl chloride (1.50 mmol) in dry CH_2_Cl_2_ (15 mL) was stirred at room temperature for 24 h. Then it was washed with HCl 1N (20 mL), saturated NaHCO_3_ (20 mL) and brine (20 mL). The organic layer was dried (Na_2_SO_4_), filtered and the solvent was evaporated to give the product as a crystal which was then purified by recrystallization from appropriate organic solvents.

*2-(Anthracen-9-yl)-1-(4-methoxyphenyl)spiro[azetidine-3,9’-xanthen]-4-one* (**2a**): Light yellow crystals from EtOAc (yield 25%); Mp: 223–225 °C; IR (CHCl_3_, cm^−1^): 1,740 (CO β-lactam); ^1^H-NMR δ (ppm): 3.64 (OMe, s, 3H) 6.29 (H-4, s, 1H), 6.51–8.82 (ArH, m, 21H); ^13^C-NMR δ (ppm) 64.3 (OMe) 82.8 (C-3), 75.6 (C-4), 116.4–152.2 (aromatic carbon), 167.3 (CO β-lactam); GC-MS m/z = 519 [M^+^]; Analysis calculated for C_36_H_25_NO_3_: C, 83.22; H, 4.85; N, 2.70%. Found: C, 83.95; H, 4.90; N, 2.82.

*2-(Anthracen-9-yl)-1-(3-methoxyphenyl)spiro[azetidine-3,9’-xanthen]-4-one* (**2b**): Yellow crystals from EtOAc (yield 54%); Mp: 212–214 °C; IR (CHCl_3_, cm^−1^): 1,755 (CO β-lactam); ^1^H-NMR δ (ppm): 3.75 (OMe, s, 3H) 6.33 (H-4, s, 1H), 6.53–8.80 (ArH, m, 21H); ^13^C-NMR δ (ppm): 55.3 (OMe) 65.6 (C-3), 75.6 (C-4), 103.5–160.4 (aromatic carbon), 167.6 (CO β-lactam); GC-MS m/z = 519 [M^+^]; Analysis calculated for C_36_H_25_NO_3_: C, 83.22; H, 4.85; N, 2.70%. Found: C, 83.95; H, 4.90; N, 2.82%.

*2-(Anthracen-9-yl)-1-(2-methoxyphenyl)spiro[azetidine-3,9’-xanthen]-4-one* (**2c**)**:** Light yellow crystals from EtOAc (yield 57%); Mp: 214–216 °C (dec.); IR (KBr, cm^−1^): 1,739 (CO β-lactam); ^1^H-NMR δ (ppm): 2.94 (OMe, s, 3H), 6.34 (H-4, s, 1H), 6.55–9.20 (ArH, m, 21H);^13^C-NMR δ (ppm): 55.4 (OMe), 66.0 (C-3), 78.5 (C-4), 112.8–152.0 (aromatic carbon), 168.0 (CO β-lactam); GC-MS m/z = 519 [M^+^]; Analysis calculated for C_36_H_25_NO_3_: C, 83.22: H, 4.85; N, 2.70%. Found: C, 83.90; H, 4.80; N, 2.81%.

*2-(Anthracen-9-yl)-1-phenylspiro[azetidine-3,9’-xanthen]-4-one* (**2d**)**:** Light yellow crystals from EtOAc (yield 63%); Mp: 238–240 °C (dec.); IR (KBr, cm^−1^): 1,758 (CO β-lactam); ^1^H-NMR δ (ppm): 6.34 (H-4, s, 1H), 6.51–8.83 (ArH, m, 22H); ^13^C-NMR δ (ppm): 65.6 (C-3), 75.4 (C-4), 115.9–152.0 (aromatic carbon), 167.5 (CO β-lactam); GC-MS m/z = 489 [M^+^]; Analysis calculated for C_35_H_23_NO_2_: C, 85.87; H, 4.74; N, 2.86%. Found: C, 85.87; H, 4.74; N 2.86%.

*2-(Anthracen-9-yl)-1-(4-chlorophenyl)spiro[azetidine-3,9’-xanthen]-4-one* (**2e**)**:** Yellow crystals from EtOAc (yield 70%); Mp: 254–256 °C (dec.); IR (KBr, cm^−1^): 1,743 (CO β-lactam); ^1^H-NMR δ (ppm): 6.30 (H-4, s, 1H), 6.52–9.06 (ArH, m, 21H); ^13^C-NMR δ (ppm): 66.0 (C-3), 75.5 (C-4), 116.0–151.9 (aromatic carbon), 167.4 (CO β-lactam); GC-MS m/z = 524 [M^+^, ^35^Cl], 526 [M^+^, ^37^Cl]; Analysis calculated for C_35_H_22_ClNO_2_: C, 80.22; H, 4.23; N 2.67%. Found: C, 80.28; H, 4.18; N, 2.53%.

*2-(Anthracen-9-yl)-1-(3-nitrophenyl)spiro[azetidine-3,9’-xanthen]-4-one* (**2f**)**:** Yellow crystals from EtOAc (yield 40%); Mp: 212–214 °C; IR (KBr, cm^−1^): 1,762 (CO β-lactam); ^1^H-NMR δ (ppm): 5.98 (H-4, s, 1H), 6.40–9.16 (ArH, m, 21H); ^13^C-NMR δ (ppm): 66.4 (C-3), 75.8 (C-4), 112.4–152.1 (aromatic carbon), 169.2 (CO β-lactam); GC-MS m/z = 534 [M^+^]; Analysis calculated for C_35_H_22_N_2_O_4_: C, 78.64; H, 4.15; N, 5.24%. Found: C, 78.63; H, 4.20; N, 5.32%.

*2-(Anthracen-9-yl)-1-(2-ethylphenyl)spiro[azetidine-3,9’-xanthen]-4-one* (**2g**)**:** White solid (yield 35%); Mp: 208–210 °C; IR (KBr, cm^−1^): 1,755 (CO β-lactam); ^1^H-NMR δ (ppm): 1.14 (t, 3H, Me, *J* = 7.1), 4.00 (CH_2_, q, 2H, *J* = 7.1), 4.92 (s, 1H, H-4), 6.30–8.74 (ArH, m, 21H); ^13^C-NMR δ (ppm): 15.1 (CH_3_), 25.8 (CH_2_), 64.5 (C-3), 75.1 (C-4), 115.9–152.2 (aromatic carbon), 168.1 (CO β-lactam); GC-MS m/z = 517 [M^+^]; Analysis calculated for C_37_H_27_NO_2_: C, 85.85; H, 5.26; N, 2.71%. Found: C, 85.83; H, 5.30; N, 2.76%.

*2-(Anthracen-9-yl)-1-(3-bromophenyl)spiro[azetidine-3,9’-xanthen]-4-one* (**2h**)**:** Yellow crystals from EtOAc (yield 55%); Mp: 222–224 °C; IR (KBr, cm^−1^): 1,755 (CO β-lactam); ^1^H-NMR δ (ppm): 6.18 (H-4, s, 1H), 6.23–8.65 (ArH, m, 21H); ^13^C-NMR δ (ppm): 66.0 (C-3), 75.6 (C-4), 115.7–151.9 (aromatic carbon), 167.7 (CO β-lactam); GC-MS m/z = 567 [M^+^, ^80^Br], 569 [M^+^, ^82^Br]; Analysis calculated for C_35_H_22_BrNO_2_: C, 73.95; H, 3.90; N, 2.46%. Found: C, 73.90; H, 3.93; N, 2.51%.

*2-(Anthracen-9-yl)-1-(2,4-dimethoxyphenyl)spiro[azetidine-3,9’-xanthen]-4-one* (**2i**)**:** Light green crystals from EtOAc (yield 69%); Mp: 180–182 °C (dec.); IR (KBr, cm^−1^): 1,739 (CO β-lactam); ^1^H-NMR δ (ppm): 2.91, 3.58 (2OMe, s, 6H) 6.16 (H-4, s, 1H), 6.17–8.18 (ArH, m, 20H); ^13^C-NMR δ (ppm): 55.4, 55.5 (s, 6H, 2 OMe) 65.9 (C-3), 78.1 (C-4), 100.2–158.2 (aromatic carbon), 167.7 (CO β-lactam); GC-MS m/z = 549 [M^+^]; Analysis calculated for C_37_H_27_NO_4_: C, 80.86; H, 4.95; N, 2.55%. Found: C, 80.03; H, 4.98; N, 2.83%.

*2-(Anthracen-9-yl)-1-(3,4-dimethoxyphenyl)spiro[azetidine-3,9’-xanthen]-4-one* (**2j**)**:** Yellow solid (yield 91%); Mp: 172–174 °C; IR (KBr, cm^−1^): 1,743 (CO β-lactam); ^1^H-NMR δ (ppm): 3.69, 3.97 (2OMe, s, 6H) 6.30 (H-4, s, 1H), 6.50–8.77 (ArH, m, 20H); ^13^C-NMR δ (ppm): 55.9, 56.1 (2 OMe) 65.6 (C-3), 75.6 (C-4), 102.3–153.0 (aromatic carbon), 166.9 (CO β-lactam); GC-MS m/z = 549 [M^+^]; Analysis calculated for C_37_H_27_NO_4_: C, 80.86; H, 4.95; N, 2.55%. Found: C, 80.81; H, 4.97; N, 2.53%.

*2-(Anthracen-9-yl)-1-cyclohexylspiro[azetidine-3,9’-xanthen]-4-one* (**2k**)**:** Orange crystals from EtOAc (yield 69%); Mp: 216–218 °C; IR (KBr, cm^−1^): 1,743 (CO β-lactam); ^1^H-NMR δ (ppm): 1.53, 1.75, 1.95, 2.22, 2.65, 3.81 (cyclohexyl, m, 11H) 5.96 (H-4, s, 1H), 6.36–9.19 (ArH, m, 17H); ^13^C-NMR δ (ppm): 24.5, 25.5, 30.0, 31.0, 56.7 (cyclohexyl) 64.5 (C-3), 72.8 (C-4), 115.9–152.4 (aromatic carbon), 169.6 (CO β-lactam); GC-MS m/z = 495 [M^+^]; Analysis calculated for C_35_H_29_NO_2_: C, 84.82; H, 5.90; N, 2.83%. Found: C, 84.81; H, 5.92; N, 2.81%.

*2-(Anthracen-9-yl)-1-(naphthalen-1-yl)spiro[azetidine-3,9’-xanthen]-4-one* (**2l**): Orange crystals from EtOAc (yield 68%); Mp: 184–186 °C (dec.); IR (CHCl_3_, cm^−1^): 1,759 (CO β-lactam); ^1^H-NMR δ (ppm): 6.45 (H-4, s, 1H), 6.61–9.8 (ArH, m, 24H); ^13^C-NMR δ (ppme) 90.3 (C-3), 74.8 (C-4), 116.7–152.1 (aromatic carbon), 168.3 (CO β-lactam); GC-MS m/z = 539 [M^+^]; Analysis calculated for C_39_H_25_NO_2_: C, 86.80; H, 4.67; N, 2.60%. Found: C, 86.52; H, 4.63; N, 2.61%.

*1-(Naphthalen-1-yl)-2-(naphthalen-2-yl)spiro[azetidine-3,9’-xanthen]-4-one* (**2m**)**:** White solid (yield 50%); Mp: 187–189 °C; IR (KBr, cm^−1^): 1,758 (CO β-lactam); ^1^H-NMR δ (ppm) 5.73 (H-4, s, 1H), 6.50–9.00 (ArH, m, 22H); ^13^C-NMR δ (ppm): 62.8 (C-3), 75.8 (C-4), 116.4–152.29 (aromatic carbon), 196.3 (CO β-lactam); GC-MS m/z = 489 [M^+^]; Analysis calculated for C_35_H_23_NO_2_: C, 85.87; H, 4.74; N, 2.86%. Found: C, 85.67; H, 4.65; N, 2.79%.

*1-(4-Methoxyphenyl)-2-(naphthalen-2-yl)spiro[azetidine-3,9’-xanthen]-4-one* (**2n**)**:** Gray solid (yield 40%); Mp: 184–186 °C; IR (CHCl_3_, cm^−1^): 1,751 (CO β-lactam); ^1^H-NMR δ (ppm): 3.81 (OMe, s, 3H) 5.21 (H-4, s, 1H), 6.64–8.33 (ArH, m, 19H); ^13^C-NMR δ (ppm): 55.5 (OMe) 64.0 (C-3), 74.6 (C-4), 113.9–151.8 (aromatic carbon), 167.0 (CO β-lactam); GC-MS m/z = 469 [M^+^]; Analysis calculated for C_32_H_23_NO_3_: C, 81.86; H, 4.94; N, 2.98%. Found: C, 81.53; H, 4.98; N, 2.83%.

### 3.5. General Procedure for the Synthesis of Bis-Polycyclic Aromatic Spiro and Nonspiro-β-Lactams ***4a–h*** and ***6a–b***

A mixture of Schiff base (1.00 mmol), triethylamine (10.00 mmol), 9*H*-xanthen-9-carboxylic acid or phenoxyacetic acid (3.00 mmol) and tosyl chloride (3.00 mmol) in dry CH_2_Cl_2_ (15 mL) was stirred at room temperature for 24 h. Then it was washed with HCl 1N (20 mL), saturated NaHCO_3_ (20 mL) and brine (20 mL). The organic layer was dried (Na_2_SO_4_), filtered and the solvent was evaporated to give the product as a crystal which was then purified by recrystallization from suitable organic solvents.

*2-(Anthracen-9-yl)-1-(4-(4-(2-(anthracen-9-yl)-4-oxospiro[azetidine-3,9’-xanthene]-1-yl)benzyl)-phenyl)spiro[azetidine-3,9’-xanthen]-4-one* (**4a**)**:** Orange solid (yield 92%); Mp: 162–164 °C (dec.); IR (KBr, cm^−1^): 1,755 (CO β-lactam); ^1^H-NMR δ (ppm): 3.76 (CH_2_, s, 2H) 6.15 (H-4, s, 2H), 6.19–8.99 (ArH, m, 42H); ^13^C-NMR δ (ppm): 40.8 (CH_2_) 65.7 (C-3), 75.4 (C-4), 115.9–152.0 (aromatic carbon), 167.3 (CO β-lactam). Analysis calculated for C_71_H_46_N_2_O_4_: C, 86.04; H, 4.68; N, 2.83%. Found: C, 86.10; H, 4.71; N, 2.80%.

*2-(Anthracen-9-yl)-1-(3-(3-(2-(anthracen-9-yl)-4-oxospiro[azetidine-3,9’-xanthene]-1-yl)benzyl) phenyl)spiro[azetidine-3,9’-xanthen]-4-one* (**4b**): Orange solid (Yield 87%); Mp: 220–222 °C (dec.); IR (KBr, cm^−1^): 1,758 (CO β-lactam); ^1^H-NMR δ (ppm): 3.78 (CH_2_, s, 2H,) 6.22 (H-4, s, 2H), 6.26–9.52 (ArH, m, 42H); ^13^C-NMR δ (ppm): 41.5 (CH_2_) 59.6 (C-3), 75.4 (C-4), 115.9–152.0 (aromatic carbon), 167.4 (CO β-lactam). Analysis calculated for C_71_H_46_N_2_O_4_: C, 86.04; H, 4.68; N, 2.83%. Found: C, 86.06; H, 4.70; N, 2.80%.

*2-(Anthracen-9-yl)-1-(4-(4-(2-(anthracen-9-yl)-4-oxospiro[azetidine-3,9’-xanthene]-1-yl)phenoxy) phenyl)spiro[azetidine-3,9’-xanthen]-4-one* (**4c**)**:** Yellow solid (Yield 75%); Mp: 242–244 °C (dec.); IR (KBr, cm^−1^): 1,755 (CO β-lactam); ^1^H-NMR δ (ppm): 6.20 (H-4, s, 2H), 6.22–8.68 (ArH, m, 42H); ^13^C-NMR δ (ppm): 65.8 (C-3), 75.5 (C-4), 115.9–153.9 (aromatic carbon), 167.0 (CO β-lactam). Analysis calculated for C_70_H_44_N_2_O_5_: C, 84.66; H, 4.47; N, 2.82%. Found: C, 84.64; H, 4.48; N, 2.80%.

*2-(Anthracen-9-yl)-1-(3-(4-(2-(anthracen-9-yl)-4-oxospiro[azetidine-3,9’-xanthene]-1-yl)phenoxy) phenyl)spiro[azetidine-3,9’-xanthen]-4-one* (**4d**)**:** Orange solid (Yield 85%); Mp: 140–142 °C; IR (KBr, cm^−1^): 1,759 (CO β-lactam); ^1^H-NMR δ (ppm): 6.15 (H-4, s, 2H), 6.40–8.79 (ArH, m, 42H). ^13^C-NMR δ (ppm): 65.8 (C-3), 75.7 (C-4), 117.5–157.4 (aromatic carbon), 167.4 (CO β-lactam). Analysis calculated for C_70_H_44_N_2_O_5_: C, 84.66; H, 4.47; N, 2.82%. Found: C, 84.63; H, 4.45; N, 2.81%.

*1,1’-(4,4’-Methylenebis(4,1-phenylene))bis(4-(anthracen-9-yl)-3-phenoxyazetidin-2-one)* (**4e**)**:** Yellow solid (Yield 80%); Mp: 146–148 °C; IR (KBr, cm^−1^): 1,755 (CO β-lactam); ^1^H-NMR δ (ppm): 3.42 (CH_2_, s, 2H) 5.85 (H-4, d, 2H, *J* = 2.8), 6.40 (H-3, d, 2H, *J* = 2.8), 6.47–9.54 (ArH, m, 36H); ^13^C-NMR δ (ppm): 40.5 (CH_2_) 62.9 (C-3), 59.1 (C-4), 115.6–157.1 (aromatic carbon), 163.3 (CO β-lactam). Analysis calculated for C_59_H_42_N_2_O_4_: C, 84.06; H, 5.02; N, 3.32%. Found: C, 84.10; H, 5.08; N, 3.35%.

*1,1’-(3,3’-Methylenebis(3,1-phenylene))bis(4-(anthracen-9-yl)-3-phenoxyazetidin-2-one)* (**4f**)**:** Orange solid (Yield 87%); Mp: 118–120 °C; IR (KBr, cm^−1^): 1,758 (CO β-lactam); ^1^H-NMR δ (ppm): 3.47 (CH_2_, s, 2H) 5.71 (H-4, d, 2H, *J* = 3.6), 6.38 (H-3, d, 2H, *J* = 3.6), 6.41–9.41 (ArH, m, 36H); ^13^C-NMR δ (ppm): 41.1 (CH_2_) 82.6 (C-3), 60.6 (C-4), 115.5–157.1 (aromatic carbon), 163.4 (CO β-lactam); Analysis calculated for C_59_H_42_N_2_O_4_: C, 84.06; H, 5.02; N, 3.32%. Found: C, 84.08; H, 5.05; N, 3.30%.

*1,1’-(4,4’-Oxybis(4,1-phenylene))bis(4-(anthracen-9-yl)-3-phenoxyazetidin-2-one)* (**4g**)**:** Orange solid (Yield 90%); Mp: 126–128 °C; IR (KBr, cm^−1^): 1,755 (CO β-lactam); ^1^H-NMR δ (ppm): 5.71 (H-4, d, 2H, *J* = 5.1), 6.20 (H-3, d, 2H, *J* = 5.1), 6.36–8.78 (m, ArH, 36H);^13^C-NMR δ (ppm): 83.1 (C-3), 59.2 (C-4), 115.6–133.0 (aromatic carbon), 167.7 (CO β-lactam). Analysis calculated for C_58_H_40_N_2_O_5_: C, 82.45; H, 4.77; N, 3.32%. Found: C, 82.41; H, 4.79; N, 3.35%.

*4-(Anthracen-9-yl)-1-(3-(4-(2-(anthracen-9-yl)-4-oxo-3-phenoxyazetidin-yl)phenoxy)phenyl)-3-phen oxyazetidin-2-one* **(4h):** Yellow solid (Yield 95%); Mp: 118–120 °C; IR (KBr, cm^−1^): 1,758 (CO β-lactam); ^1^H-NMR δ (ppm): 5.75 (H-4, d, 2H, *J* = 3.8), 6.25 (H-3, d, 2H, *J* = 3.8), 6.42–8.79 (ArH, m, 36H); ^13^C-NMR δ (ppm): 83.1 (C-3), 65.4 (C-4), 107.7–157.4 (aromatic carbon), 163.5 (CO β-lactam). Analysis calculated for C_58_H_40_N_2_O_5_: C, 82.45; H, 4.77; N, 3.32%. Found: C, 82.43; H, 4.75; N, 3.30%.

*1,1’-(1,4-Phenylene)bis(4-(anthracen-9-yl)-3-phenoxyazetidin-2-one)* (**6a**)**:** Gray solid (Yield 96%); Mp: 178–180 °C (dec.); IR (KBr, cm^−1^): 1,751 (CO β-lactam); ^1^H-NMR δ (ppm): 5.58 (H-4, d, 2H, *J* = 3.9), 6.30 (H-3, d, 2H, *J* = 3.9), 6.33–8.82 (ArH, m, 32H);^13^C-NMR δ (ppm): 83.0 (C-3), 59.1 (C-4), 114.6–156.8 (aromatic carbon), 163.0 (CO β-lactam). Analysis calculated for C_52_H_36_N_2_O_4_: C, 82.96; H, 4.82; N, 3.72% Found: C, 82.95; H, 4.81; N, 3.70%.

*1,1’-(1,4-Phenylene)bis(2-(anthracen-9-yl)spiro[azetidine-3,9’-xanthen]-4-one)* (**6b**)**:** Orange solid (Yield 85%); Mp: 186–188 °C (dec.); IR (KBr, cm^−1^): 1,751 (CO β-lactam); ^1^H-NMR δ (ppm): 6.27 (H-4, s, 2H), 6.30–8.99 (ArH, m, 38H); ^13^C-NMR δ (ppm): 59.7 (C-3), 75.2 (C-4), 115.9–152.0 (aromatic carbon), 167.0 (CO β-lactam). Analysis calculated for C_64_H_40_N_2_O_4_: C, 85.31; H, 4.47; N, 3.11%. Found: C, 85.30; H, 4.45; N, 3.15%.

## 4. Conclusions 

This article describes for the first time the synthesis and characterization of some examples of mono-and bis-spiro- and nonspiro-β-lactams bearing a polycyclic aromatic moiety by reaction of polycyclic aromatic imines and two ketenes derived from 9*H*-xanthene-9-carboxylic acid and phenoxyacetic acid. These ketenes were prepared *in situ* with triethylamine and *p*-toluenesulfonyl chloride.

## Figures and Tables

**Table 1:** Structures of spiro-β-lactams.

Entry	Product	Yield (%) ^a^	Entry	Product	Yield (%) ^a^
1	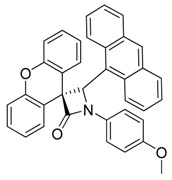 **2a**	25	6	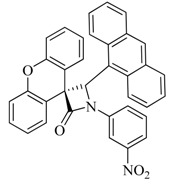 **2f**	40
2	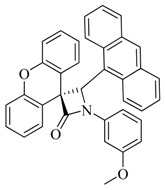 **2b**	54	7	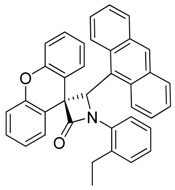 **2g**	35
3	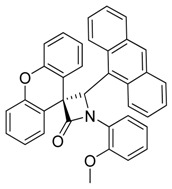 **2c**	51	8	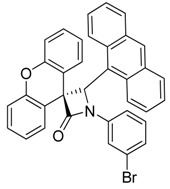 **2h**	53
4	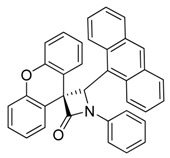 **2d**	63	9	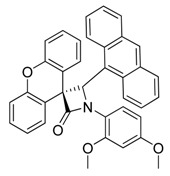 **2i**	70
5	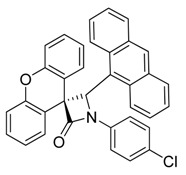 **2e**	70	10	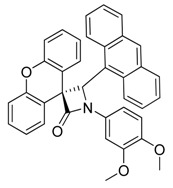 **2j**	91
11	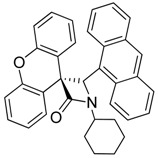 **2k**	69	13	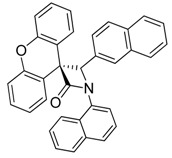 **2m**	50
12	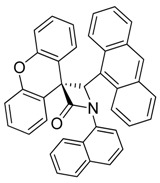 **2l**	68	14	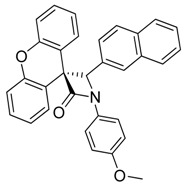 **2n**	40

^a^ Isolated yield of pure products.

**Table 2:** Structures of bis-spiro and bis-nonspiro-β-lactams.

Entry	Product	Yield(%)^a^	Entry	Product	Yield(%)^a^
1	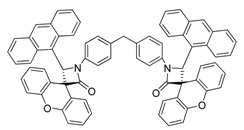 **4a**	92	6	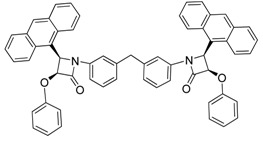 **4f**	87
2	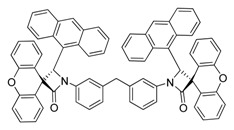 **4b**	87	7	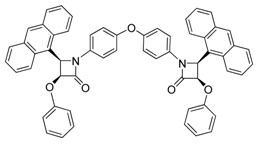 **4g**	90
3	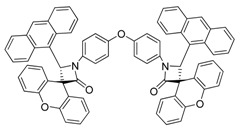 **4c**	96	8	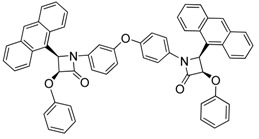 **4h**	95
4	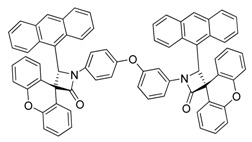 **4d**	85	9	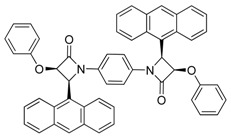 **6a**	96
5	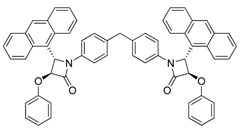 **4e**	80	10	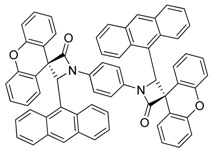 **6b**	85

^a^ Isolated yields of pure products.
